# Changes in Gait Performance in Stroke Patients after Taping with Scapular Setting Exercise

**DOI:** 10.3390/healthcare8020128

**Published:** 2020-05-08

**Authors:** Shin Jun Park, Seunghue Oh

**Affiliations:** 1 Department of Physical Therapy, Gangdong University, 278, Daehak-gil, Gamgok-myeon, Eumseong-gun, Chungcheongbuk-do 27600, Korea; p3178310@gangdong.ac.kr; 2 Department of Physical Therapy, Graduate School, Dankook University, 119, Dandae-ro, Dongnam-gu, Cheonan-si, Chungnam 330-714, Korea

**Keywords:** gait parameter, Kinesio taping, scapular setting exercise, stroke

## Abstract

The purpose of this study was to investigate the effects of combined taping with scapular setting exercise on the gait performance of stroke patients. Twenty stroke patients were randomly allocated to two groups: the taping with scapular setting exercise (TSSE) group (*n* = 10) and scapular setting exercise (SSE) group (*n* = 10). Intervention was performed for one week, and pre- and postintervention results for TSSE and SSE were compared. Outcomes were determined using the inertia measurement unit, which can measure spatiotemporal gait parameters, and using the timed up-and-go test. Two-way repeated analysis was used to compare pre- and postintervention results. In the TSSE group, intervention significantly improved cadence, gait speed, stride length, step length, gait cycle, swing phase duration, double support duration, and timed up-and-go test results more than in the SSE group. TSSE was found to improve all spatiotemporal gait parameters examined; thus, we recommend TSSE be considered as an intervention to improve gait parameters in stroke patients.

## 1. Introduction

Many stroke patients have problems with motor coordination and voluntary movement due to motor paralysis and motor impairment [1,2]. The most evident motor impairment in stroke patients is hemiparesis [3], which impairs walking ability in more than 80% of stroke patients [4]. Representative features of hemiparetic gait include slow walking speed, prolonged stance duration on the nonparetic side, increased double support time, and an asymmetric gait [2,5,6]. For this reason, improving gait performance after stroke is essential to enhance quality of life [7].

Walking is a complex and coordinated task, and stable walking is always accompanied by proper posture control and balance [8,9]. Thus, gait control requires the coordination of not only lower limbs but also of trunk and upper limb movements [10,11,12,13]. Additionally, upper limbs influence gait pattern [10], and upper limb involvement during gait alters lower limb muscle activation [13]. In particular, scapular movement is an essential component of upper limb movements [14], and scapular stabilizers can affect the gait ability of patient with hemiparesis [15], presumably because they alter gait pattern. However, scapular stabilizers are often impaired in stroke patients, and thus they cannot be used to assist gait properly [16,17]. These findings suggest that limited scapular movement is likely to induce a hemiparetic gait, and that intervention targeting scapular stabilizer muscles may be important for improving gait function in stroke patients [15].

For patients with impaired gait due to central nervous system injuries, it is necessary to train their gait for locomotion [18]. Several studies reported that upper extremity exercises had resulted in a better outcome for gait performance because rhythmic arm movements induced by upper limb exercise influence body stabilization and voluntary arm swings during gait induce corticospinal tract activity and reflex reactions, and thereby induce lower limb muscle activities [19]. The scapular setting exercise (SSE) is used to promote correct scapula positioning and stabilizing movement control during standing and walking [20,21,22], and the taping method is widely used to improve joint movement and balance [23]. In spite of the effect of SSE and taping intervention, the study of combined taping with scapular setting exercise (TSSE) has not been investigated.

In the present study, we investigate the effect of TSSE on the gait performance of stroke patients for more effective means of intervention than SSE.

## 2. Method

### 2.1. Participants

Twenty stroke patients admitted to a rehabilitation hospital in Gyeonggi-do, Korea were included in this study. All twenty volunteered to participate and were given full details of the study and its requirements. The inclusion criteria were as follows: (1) stroke onset during the previous 6 months, (2) Brunnstrom stage ≤ III on the upper extremity, (3) a Korean Mini-Mental State Examination score of ≥ 24, (4) the ability to walk independently for ≥ 10 m, (5) no upper or lower extremity fracture, and (6) no skin condition affecting tape placement. The exclusion criteria applied were as follows: (1) the presence of a sensory defect by the pin-prick test, (2) severe shoulder pain, and (3) grade 0 as determined by the upper extremity manual muscle tests. This study was approved by the Yong-In University Institutional Research Review Committee (2-1040966-AB-N-01-20-1812-HSR-125-10).

### 2.2. Study Design and Process

Twenty subjects were randomly assigned to the TSSE group (*n* = 10) or the SSE group (*n* = 10) using a computer-generated random number table. The experimental group (*n* = 10) received a scapular setting exercise with taping, and the control group (*n* = 10) received only a scapular setting exercise. Scapular setting exercise was performed by one physiotherapist who had significant experience regarding scapular setting exercise and taping. The taping application was applied by the same physiotherapist. All subjects received intervention for 1 week. Gait analysis was performed in both groups at pre- and postintervention.

The sample size of this study was determined using G-power software (G* Power 3.1.9.2, Heinrich-Heine-Universität, Düsseldorf, Germany). Based on a pilot study of 5 subjects, partial η^2^ was 0.207 and total required sample size (level of 0.05, power of 0.80, and effect size of 0.51) was 10. 

### 2.3. Measurements

To observe the effect of both TSSE and SSE intervention, we obtained the following spatiotemporal gait parameters, that is, cadence; gait speed; stride length; step length; and durations of the stance phase, swing phase, double support phase, and single support phase, using the inertia measurement unit (IMU) system (G-walk, BTS Inc, Milan, Italy), which was placed on the 5th lumbar vertebra and fixed in position using a Velcro strap. With a subject in a standing position, the monitor was checked, and the start button was pressed. At this time, the signal “duration of the stabilization phase” disappeared, and the subject walked 8 m at a self-determined speed. When the trailing foot of the subject crossed the 8 m finish line, the stop button was pressed. In addition, the timed up-and-go (TUG) test was used to assess dynamic gait ability.

#### Timed Up-and-Go (TUG) Test

The equipment used was a chair with a backrest, an Iphone 7 plus (Apple Inc., Cupertino, California, USA), and measured distance was 3 m. With a subject seated on the chair, a start signal was given, and the subject then stood up and walked 3 m directly in front of the chair. TUG times were defined as the sum of times taken to rise from the chair, walk the 3 m, turn around, walk back to the chair, and sit down [24]. Times were recorded to 0.01 sec, and the test was performed in triplicate. Average values were used in the analysis. The test-retest reliability of the TUG test was high (0.95) [25].

### 2.4. Intervention Method

#### 2.4.1. Scapular Setting Exercise (SSE)

All 20 study subjects underwent TSSE or SSE on paretic sides. SSE was used to increase upward and external rotation and posterior or anterior tilt of the scapula [22] with a subject sitting on a treatment table with both feet on the floor. The therapist placed his index finger and thumb of the right hand on the lateral and medial border of the scapula and fixed the inferior angle with the first web space. The four fingers of the left hand were wrapped around the pectoralis major muscle with the thumb placed on the axilla. The right hand was used to move the scapula in an upward and downward rotation, adduction, and abduction to enable the subject to recognize the direction of movement. In addition, the therapist compressed the scapula using his palm and applied distraction to lift the scapular medial border. The intervention was performed 10 times for approximately 30 min with a 30-s resting period for 7 consecutive days.

#### 2.4.2. Kinesio Taping Method

The TSSE group received scapula-upward taping, which was performed to increase upward and external rotations and posterior tilt of the scapula. The Kinesio tape used was a 5 cm wide adhesive tape (TS Co., Gyeonggi-Do, South Korea). Four taping straps were used. The first tape positioned was attached from the acromion to the cervical spine; the second, from the upper trapezius to the scapular inferior angle; the third, from the scapular inferior angle to the posterior deltoid; and the fourth, from the scapular spine to the 12th thoracic vertebra [26]. Taping was applied with 50–75% elasticity immediately after SSE and reattached after 24 h. Remaining tape adhesive was removed by applying lotion to skin. Placebo taping was performed in the SSE group in the same way but using nonelastic surgical tape (3M, Maplewood, MN, USA).

### 2.5. Statistical Analyses

Data were analyzed using SPSS 20.0 software (SPSS, Inc., Chicago, IL, USA). First, we conducted the Shapiro–Wilk test to confirm the general characteristics of subjects. Then, the independent t-test was used to confirm subject homogeneity. Finally, two-way repeated measures analysis was applied to determine whether there was a statistically significant difference in change of gait parameters and TUG test to compare pre- and postintervention results. Within-subject variables were compared pre- and postintervention (time). The between-subject variables were compared SSE group and TSSE group (group-by-time). If significant differences were observed main effects or group-by-time interaction, t-test was performed for post hoc test. The level of significance was set at α = 0.05.

## 3. Result

The general characteristics of subjects are shown in Table 1. No significant difference in general characteristics was observed between the SSE and TSSE groups.

The TSSE group showed significant improvements in all gait parameters (cadence, gait speed, stride length, step length, gait cycle, swing phase duration, double support duration, and TUG test results) at postintervention compared with preintervention (Table 2). Additionally, the *t*-values are also greater than 1.96 and provide the significance of the results [27]. In contrast, the SSE group showed significant difference was observed at postintervention in gait speed only compared with pre-intervention. Except gait speed, there are no significant differences in SSE group at postintervention compared with preintervention. The comparison of group-by-time interaction for gait parameter showed there is no significant difference. 

## 4. Discussion

In this study, we investigated the changes in gait performance in stroke patients after TSSE, compared with SSE. From the experimental comparison, we found substantial improvements in gait performance post intervention in the TSSE group, as compared with the SSE group.

Previous studies report that stroke patients have shorter step lengths and longer double stance and longer swing phases of the affected side [5,6,28]. Guzik et al. reported that gait symmetry may be an important factor of level of gait control in poststroke patients [29]. Our results showed the characteristics of a symmetric gait pattern. Specifically, in TSSE group when single support duration decreased, double support duration of the affected side increased, which indicates the gait pattern became more symmetric. Ortega et al. reported that arm swing movements improve the symmetrical use of upper limbs and trunk alignment [30]. Other studies have also reported that upper extremity exercises increase the activities of leg muscles in normal adults and in patients with neuropathy [31]. These results suggest rhythmic arm movements influence body stabilization by harmonizing control of body angular momentum. In addition, recent studies have reported that voluntary arm swings during gait induce corticospinal tract activity and reflex reactions, and thereby induce lower limb muscle activities [19]. The results of these prior studies concur with our findings, and suggest the significant improvements in gait performance. According to these knowledges, TSSE intervention improved arm swing movement and induced symmetrical use of upper limbs and trunk alignment. Therefore, TSSE induced positive changes in gait performance.

Stephenson et al. reported that walking speed was reduced when arm movement was restricted during walking [32], and that increased arm swing in stroke patients led to increased stride length and transverse pelvic and thoracic rotations [11]. In addition, Bovonsunthonchai et al. reported that coordination of affected shoulder and contralateral hip movements were associated with gait speed [33]. The elastic taping applied in this study was attached to increase scapular movement by facilitating upward rotation of the scapular. We speculate increased arm swing due to taping increased gait speed by promoting coordination of the nonaffected leg.

Though improvements in gait parameters were observed in the SSE group, they were not significant, which may have been because the duration of SSE intervention was too short. In a previous study, scapulohumeral rehabilitation in subacute stroke patients required 6 weeks to improve Trunk Impairment Scale, modified Barthel Index, Sitting Balance Scale, and Trunk Control Test scores [34]. On the other hand, several taping-related studies have reported [35,36] that taping has an immediate effect. Thus, we believe that the 1-week intervention period explains the significant differences observed between the TSSE and SSE groups.

In the present study, we used placebo taping to account for an active placebo effect. Moreover, the active placebo used in the present study closely mimicked the real taping method. The protocol that used the same total number of active SSE treatments was performed in both groups to distinguish the effects of real and sham taping. Other studies that applied the placebo taping method to stroke patients’ shoulders had no effect on joint position sense [37].

The present study has a number of limitations that warrant consideration. First, it is limited by its small sample size. Second, our intervention period was shorter than that used in a previous study [34]. However, we found intervention produced significant improvements in cadence, gait speed, and stride and step length, and in gait cycle, stance and swing phase, and double support durations. Third, the mechanism responsible for the potentiating action of upper limb movement was not determined. However, we suggest biomechanical and neurophysiologic mechanisms may underlie the effect of upper limb movements on the symmetry of lower limb movements.

## 5. Conclusions

The result of this study showed changes in gait performance in stroke patients after TSSE compared with SSE. By comparing the TSSE with the SSE intervention, we found that TSSE intervention improved gait performance more than SSE. Thus, TSSE intervention would result in better outcome of gait function for stroke patients.

## Figures and Tables

**Table 1:** Subject characteristics.

Categories	SSE Group (*n* = 10)	TSSE Group (*n* = 10)	*p*
Gender (male/female)	5/5	8/2	0.160
Etiology (infarction/hemorrhage)	4/6	6/4	1.000
Paretic side (left/right)	6/4	5/5	0.653
Age (years)	63.10 ± 8.07	63.40 ± 7.79	0.934
Height (cm)	159.10 ± 7.48	164.20 ± 6.97	0.132
Weight (kg)	63.40 ± 8.60	65.60 ± 5.56	0.506
Disease duration (month)	4.70 ± 0.67	4.00 ± 1.33	0.156
K-MMSE (point)	27.20 ± 1.03	26.90 ± 1.28	0.572

SSE: scapular setting exercise, TSSE: taping with scapular setting exercise. K-MMSE, Korean Mini-Mental State Examination.

**Table 2:** Change in gait parameters and timed up-and-go (TUG) test.

Variable	Group	Pre Intervention	Post Intervention	*t*	*p*
Cadence (step/min)	SSE	88.01 ± 17.32	90.38 ± 18.03	–1.828	0.006 **
	TSSE	86.56 ± 22.71	92.13 ± 19.59^1^	–2.563^2^	
Gait speed (m/s)	SSE	0.81 ± 0.28	0.87 ± 0.28	–3.779^2^	0.001 **
	TSSE	0.83 ± 0.30	0.92 ± 0.26^1^	–3.457^2^	
Stride length (m)	SSE	1.10 ± 0.22	1.12 ± 0.19	–1.901	0.011 *
	TSSE	1.10 ± 0.22	1.19 ± 0.23^1^	–2.399^2^	
Gait cycle duration (sec)	SSE	1.42 ± 0.32	1.37 ± 0.30	1.346	0.038 *
	TSSE	1.50 ± 0.49	1.39 ± 0.50^1^	2.558^2^	
Step length (%)	SSE	47.24 ± 3.49	47.27 ± 3.59	–0.039	0.048 *
	TSSE	47.49 ± 2.61	49.24 ± 1.50^1^	–2.345^2^	
Stance phase duration (%)	SSE	59.55 ± 3.44	62.59 ± 8.17	–0.965	0.041 *
	TSSE	59.30 ± 4.48	63.84 ± 6.65^1^	–2.320^2^	
Swing phase duration (%)	SSE	40.45 ± 3.44	37.41 ± 8.17	0.965	0.035 *
	TSSE	40.80 ± 4.61	36.16 ± 6.65^1^	2.379^2^	
Double support duration (%)	SSE	14.08 ± 8.25	13.55 ± 7.22	0.311	0.034 *
	TSSE	18.01 ± 9.60	14.08 ± 8.43^1^	2.322^2^	
Single support duration (%)	SSE	32.54 ± 10.00	35.56 ± 9.38	–1.236	0.049 *
	TSSE	31.88 ± 10.63	37.41 ± 7.16^1^	–2.474^2^	
TUG test (sec)	SSE	16.89 ± 4.25	15.84 ± 3.57	1.571	0.025 *
	TSSE	17.83 ± 3.05	15.97 ± 3.10^1^	2.865	

Results are presented as means ± SDs, * *p* < 0.05, as determined by two-way repeated measures analysis of variance (* *p* < 0.05, ** *p* < 0.01). ^1^ Significant different after intervention (*p* < 0.05). ^2^ The *t*-values for variables are greater than 1.96.

## References

[1] Higginson J., Zajac F., Neptune R.R., Kautz S., Delp S. (2006). Muscle contributions to support during gait in an individual with post-stroke hemiparesis. J. Biomech..

[2] Muto T., Herzberger B., Hermsdorfer J., Miyake Y., Poppel E. Interactive gait training device “Walk-Mate” for hemiparetic stroke rehabilitation. Proceedings of the 2007 IEEE/RSJ International Conference on Intelligent Robots and Systems.

[3] Umphred D.A. (1994). Fisioterapia Neurológica.

[4] Duncan P.W., Zorowitz R., Bates B., Choi J.Y., Glasberg J.J., Graham G.D., Katz R.C., Lamberty K., Reker D. (2005). Management of adult stroke rehabilitation care: A clinical practice guideline. Stroke.

[5] Olney S.J., Richards C. (1996). Hemiparetic gait following stroke. Part I: Characteristics. Gait Posture.

[6] Algurén B., Lundgren-Nilsson Å., Sunnerhagen K.S. (2010). Functioning of stroke survivors—A validation of the ICF core set for stroke in Sweden. Disabil. Rehabil..

[7] Dobkin B.H. (2005). Rehabilitation after stroke. N. Engl. J. Med..

[8] Takakusaki K. (2013). Neurophysiology of gait: From the spinal cord to the frontal lobe. Mov. Disord..

[9] Massion J. (1992). Movement, posture and equilibrium: Interaction and coordination. Prog. Neurobiol..

[10] Jackson K., Joseph J., Wyard S. (1983). The upper limbs during human walking. Part 2: Function. Electromyogr. Clin. Neurophysiol..

[11] Ford M.P., Wagenaar R.C., Newell K.M. (2007). Phase manipulation and walking in stroke. J. Neurol. Phys. Ther..

[12] Visintin M., Barbeau H. (1994). The effects of parallel bars, body weight support and speed on the modulation of the locomotor pattern of spastic paretic gait. A preliminary communication. Spinal Cord.

[13] Huang H.J., Ferris D.P. (2004). Neural coupling between upper and lower limbs during recumbent stepping. J. Appl. Physiol..

[14] van Andel C., van Hutten K., Eversdijk M., Veeger D., Harlaar J. (2009). Recording scapular motion using an acromion marker cluster. Gait Posture.

[15] Kim J., Lee B., Lee J. (2017). Effect of scapular stabilization exercise during standing on upper limb function and gait ability of stroke patients. J. Neurosci. Rural Pract..

[16] Hwang Y.-I., An D.-H. (2015). Immediate effects of an elastic arm sling on walking patterns of chronic stroke patients. J. Phys. Ther. Sci..

[17] Oujamaa L., Relave I., Froger J., Mottet D., Pelissier J.-Y. (2009). Rehabilitation of arm function after stroke. Literature review. Ann. Phys. Rehabil. Med..

[18] Canning C.G., Ada L., Paul S.S. (2006). Is automaticity of walking regained after stroke?. Disabil. Rehabil..

[19] Ogawa T., Sato T., Ogata T., Yamamoto S.I., Nakazawa K., Kawashima N. (2015). Rhythmic arm swing enhances patterned locomotor-like muscle activity in passively moved lower extremities. Physiol. Rep..

[20] Mottram S. (1997). Dynamic stability of the scapula. Man. Ther..

[21] Ebaugh D.D., McClure P.W., Karduna A.R. (2005). Three-dimensional scapulothoracic motion during active and passive arm elevation. Clin. Biomech..

[22] Awad A., Shaker H., Shendy W., Fahmy M. (2015). Effect of shoulder girdle strengthening on trunk alignment in patients with stroke. J. Phys. Ther. Sci..

[23] Rojhani-Shirazi Z., Amirian S., Meftahi N. (2015). Effects of ankle kinesio taping on postural control in stroke patients. J. Stroke Cerebrovasc. Dis..

[24] Podsiadlo D., Richardson S. (1991). The timed “Up & Go”: A test of basic functional mobility for frail elderly persons. J. Am. Geriatr. Soc..

[25] Ng S.S., Hui-Chan C.W. (2005). The timed up & go test: Its reliability and association with lower-limb impairments and locomotor capacities in people with chronic stroke. Arch. Phys. Med. Rehabil..

[26] Kim B.-J., Lee J.-H. (2015). Effects of scapula-upward taping using kinesiology tape in a patient with shoulder pain caused by scapular downward rotation. J. Phys. Ther. Sci..

[27] Asif M., Jameel A., Hussain A., Hwang J., Sahito N. (2019). Linking Transformational Leadership with Nurse-Assessed Adverse Patient Outcomes and the Quality of Care: Assessing the Role of Job Satisfaction and Structural Empowerment. Int. J. Environ. Res. Public Health.

[28] Mauritz K.H. (2002). Gait training in hemiplegia. Eur. J. Neurol..

[29] Guzik A., Drużbicki M., Przysada G., Kwolek A., Brzozowska-Magoń A., Sobolewski M. (2017). Relationships between walking velocity and distance and the symmetry of temporospatial parameters in chronic post-stroke subjects. Acta Bioeng. Biomech..

[30] Ortega J.D., Fehlman L.A., Farley C.T. (2008). Effects of aging and arm swing on the metabolic cost of stability in human walking. J. Biomech..

[31] Behrman A.L., Harkema S.J. (2000). Locomotor training after human spinal cord injury: A series of case studies. Phys. Ther..

[32] Stephenson J.L., De Serres S.J., Lamontagne A. (2010). The effect of arm movements on the lower limb during gait after a stroke. Gait Posture.

[33] Bovonsunthonchai S., Hiengkaew V., Vachalathiti R., Vongsirinavarat M., Tretriluxana J. (2012). Effect of speed on the upper and contralateral lower limb coordination during gait in individuals with stroke. Kaohsiung J. Med. Sci..

[34] Dell’Uomo D., Morone G., Centrella A., Paolucci S., Caltagirone C., Grasso M.G., Traballesi M., Iosa M. (2017). Effects of scapulohumeral rehabilitation protocol on trunk control recovery in patients with subacute stroke: A pilot randomized controlled trial. NeuroRehabilitation.

[35] Wang J.-S., Um G.-M., Choi J.-H. (2016). Immediate effects of kinematic taping on lower extremity muscle tone and stiffness in flexible flat feet. J. Phys. Ther. Sci..

[36] Fayson S.D., Needle A.R., Kaminski T.W. (2013). The effects of ankle Kinesio^®^ taping on ankle stiffness and dynamic balance. Res. Sports Med..

[37] dos Santos G.L., Souza M.B., Desloovere K., Russo T.L. (2017). Elastic tape improved shoulder joint position sense in chronic hemiparetic subjects: A randomized sham-controlled crossover study. PLoS ONE.

